# Outcomes of advance care directives after admission to a long-term care home: DNR the DNH?

**DOI:** 10.1186/s12877-021-02699-5

**Published:** 2022-01-03

**Authors:** Rhéda Adekpedjou, George A. Heckman, Paul C. Hébert, Andrew P. Costa, John Hirdes

**Affiliations:** 1 Carrefour de l’innovation et de l’évaluation en santé, Centre Hospitalier de l’Université de Montréal, Montréal, Canada; 2 School of Public Health Sciences, University of Waterloo, 200 University Avenue West, Waterloo, Ontario N2L 3G1 Canada; 3 Schlegel Research Institute for Aging, Waterloo, Canada; 4 McMaster University, Hamilton, Canada

**Keywords:** Advance care planning, Long-term care homes, Nursing homes, interRAI

## Abstract

**Background:**

Residents of long-term care homes (LTCH) often experience unnecessary and non-beneficial hospitalizations and interventions near the end-of-life. Advance care directives aim to ensure that end-of-life care respects resident needs and wishes.

**Methods:**

In this retrospective cohort study, we used multistate models to examine the health trajectories associated with Do-Not-Resuscitate (DNR) and Do-Not-Hospitalize (DNH) directives of residents admitted to LTCH in Ontario, Alberta, and British Columbia, Canada. We adjusted for baseline frailty-related health instability. We considered three possible end states: change in health, hospitalization, or death. For measurements, we used standardized RAI-MDS 2.0 LTCH assessments linked to hospital records from 2010 to 2015.

**Results:**

We report on 123,003 LTCH residents. The prevalence of DNR and DNH directives was 71 and 26% respectively. Both directives were associated with increased odds of transitioning to a state of greater health instability and death, and decreased odds of hospitalization. The odds of hospitalization in the presence of a DNH directive were lowered, but not eliminated, with odds of 0.67 (95% confidence interval 0.65–0.69), 0.63 (0.61–0.65), and 0.47 (0.43–0.52) for residents with low, moderate and high health instability, respectively.

**Conclusion:**

Even though both DNR and DNH orders are associated with serious health outcomes, DNH directives were not frequently used and often overturned. We suggest that policies recommending DNH directives be re-evaluated, with greater emphasis on advance care planning that better reflects resident values and wishes.

## Background

In Canada, long-term care homes (LTCH) designate residential facilities that provide 24-h nursing and personal support to people who can no longer live independently in the community [1]. The period following LTCH admission is one during which many residents experience greater health instability, are hospitalized, or die [2]. LTCH residents are at increased risk of acute care utilization, with about one third being hospitalized yearly, during which they often undergo multiple diagnostic tests and procedures [3]. Many of these acute care visits and treatments are potentially avoidable, frequently unnecessary, and often discordant with a resident’s care wishes and preferences [4]. Indeed, older adults often focus on quality of life, function, and symptom control, and most prefer to die at home rather than in hospital [5].

Advance care planning is a process that supports residents, their families, and health care professionals make decisions about future medical care that reflect resident goals, values and preferences [6]. If done well, advance care planning protects resident autonomy at the end-of-life, even in the presence of cognitive impairment [6, 7]. In Canada, LTCH homes usually codify the outcomes of advance care planning in medical orders such as Do-Not-Hospitalize (DNH) and Do-Not-Resuscitate (DNR), though policies and regulations surrounding related care processes vary among individual Canadian provinces and territories [8, 9]. Such directives are associated with fewer hospitalisations and hospital days, death in acute care, and use of life-sustaining treatments such as cardiopulmonary resuscitation, and with a marginal impact on survival time. They are also associated with an increased probability of dying in the LTCH [7, 8].

Recent population-based data from in Ontario, Canada LTCH shows that while frailty-related health instability of newly admitted residents was associated with the presence of DNR and DNH directives, this very instability remained associated with an increased risk of hospitalisation during the first 90 days after admission [3, 8]. These findings suggest that while the presence of a DNR or DNH directive identifies more unstable and frailer LTCH residents potentially less likely to benefit from hospitalization, the DNH directive may be challenging to implement in actual practice. It is thus important to understand how admission health status and directives such as DNR and DNH relate to resident transitions to different health states, particularly during the period following admission to LTCH, in order to ensure that residents receive care that is concordant with their wishes. The aim of the present study is to understand the association of DNR and DNH directives, adjusted for baseline health instability, on subsequent transitions to different health states for newly admitted residents to LTCH in three Canadian provinces.

## Methods

### Study design

We conducted a retrospective cohort study of newly admitted LTCH residents from January 1st 2010 to March 31st 2015 in British Columbia, Alberta, and Ontario, Canada. We obtained approval from the University of Waterloo Office of Research Ethics (ORE# 18228). We complied with the Declaration of Helsinki. We applied RECORD (Reporting of Studies Conducted Using Observational Routinely Collected Health Data) and STROBE (Strengthening the Reporting of Observational Studies in Epidemiology) guidelines to report our findings [10, 11].

### Data sources

We used the following administrative data sets (a) the Complex Continuing Care Reporting System (CCRS) based on the interRAI Resident Assessment Instrument-Minimum Data Set (RAI-MDS) 2.0 for LTCH, (b) the Discharge Abstract Database (DAD) which tracks acute hospital admissions and (c) the National Ambulatory Care Reporting System (NACRS) which tracks emergency department visits. We obtained these from the Canadian Institute for Health Information, which exerts strict controls over data quality, validity, and integrity of each of these data sources [12–14]. These data sets were linked using individual resident’s unique Provincial health insurance identifiers.

The RAI-MDS 2.0 is a standard assessment mandated for use in 8 of 13 Canadian provinces and territories. The instrument is administered by trained assessors within 14 days of admission, with follow-up assessments completed every 90 days, or earlier in the event of major clinical changes [1]. The instrument contains over 300 clinical variables to support care planning, outcome measurement, quality improvement and resource allocation, and multiple embedded scales such as the Activities of Daily Living Hierarchy Scale, the Instrumental Activities of Daily Living Scale [15], the Changes in Health, End-Stage Disease, Signs, and Symptoms Scale (CHESS) [16, 17], the Depression Rating Scale [18, 19], and the Cognitive Performance Scale [20–22]. The presence of DNR and DNH directives in the resident chart is also recorded in the RAI-MDS 2.0. Reliability and validity of the RAI 2.0 and associated items, scales and algorithms have been well-established by several international studies [23–25]. Likewise, the reliability of that data captured in the DAD and NACRS has also been established (2, 3).

### Study cohort

**Fig. 1:**
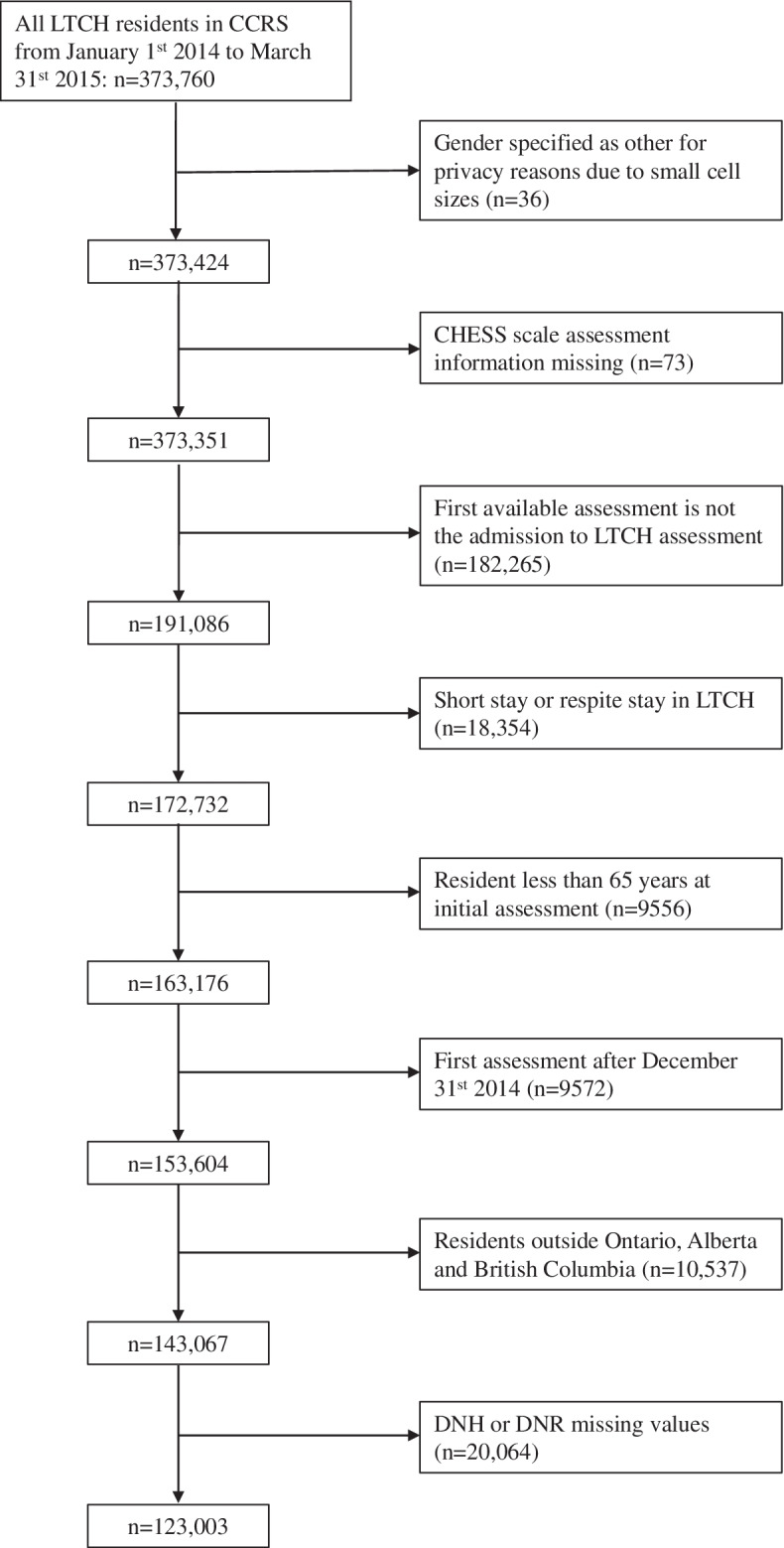
Derivation of the analytical cohort

We used data from January 1st 2010 to March 31st 2015 targeting LTCH residents in Ontario, Alberta and British Columbia. These provinces were included because their RAI-MDS 2.0 information systems were well-established during our study period. We included linked residents (i.e., whose information was contained in multiple data sources) if they had (a) at least 1 follow-up assessment at 90 days after admission to the LTCH (b) or a date of discharge or death within 90 days of admission to the LTCH. The initial cohort included 373,760 residents from across Canada (see Fig. 1). We excluded residents if: 1) gender was “Other” because small cell sizes would create privacy concerns (*n* = 36), 2) the assessment or entry date was missing, 3) CHESS scale assessment information was incomplete (*n* = 73), 4) the first available assessment is not the admission to LTCH assessment for the resident and which antedates the study period (*n* = 182,265), 5) the stay in a LTCH was purely short stay to provide the family caregiver with temporary respite (*n* = 18,354), 6) the resident was aged less than 65 years old at time of initial assessment (*n* = 9556), 7) first assessments were after Dec 31st 2014 to allow for 90 day follow-up to March 31st 2015 (*n* = 9572), 8) residents were from provinces other than Ontario, Alberta and British Columbia (*n* = 10,537), or 9) DNH or DNR values were missing on the initial assessment (*n* = 20,064).

### Outcomes of interests

We tracked five types of transitions in the 90-day following admission to the LTCH: 1) transition to a different state health instability; 2) discharge from the LTCH; 3) transfer to other care settings (e.g., other LTCH, hospice, home); 4) hospitalization, and 5) death. Health instability was measured with the CHESS (see Table 1). The CHESS scale rates health risk related to acutely decompensated frailty with scores from 0 (low instability) to 5 (high instability), and higher scores predict hospitalization and mortality among LTCH residents [16, 17]. We defined 3 states of health instability corresponding to low (CHESS 0), medium (CHESS 1–2) and high (CHESS 3–5) instability [16, 17]. We used 0 as a distinctive first state (rather than 0–1) because 0 designates the absence of any indicators of health instability, a clinically relevant break point. We ascertained hospitalizations, death, and discharge to another setting using the relevant CCRS, DAD, and NACRS variables [12–14].

**Table 1:** The CHESS Scale

The CHESS scale: The RAI MDS 2.0 is completed by trained assessors. These make use of all possible sources of information to assess a LTCH resident. CHESS scale scores range from 0 (no health instability) to 5 (very high health instability) based on the following:	
• 1 point each for recent (within 2 weeks) decline in Activities of Daily Living and Cognitive Performance;	
• Up to 2 points for the presence of any of the following: insufficient fluid intake, peripheral edema, shortness of breath, vomiting, weight loss, decrease in food eaten;	
• 1 point based on the clinical judgement of a physician that a resident has at most 6 months to live.	

### Statistical methods

We used methods previously applied to data of this type by Cook and colleagues and summarize them briefly here [26, 27]. Multistate transition analysis, based on discrete time nonhomogeneous Markov chain models, is a powerful tool to examine longitudinal changes in multiple health status outcomes over time and identify factors that influence these changes, thus allowing for the examination of competing risks in models where different outcomes (e.g., death and hospital admission) may be affected by similar risk factors [27]. Sample description with descriptive statistics is an intermediate step of Multistate transition analysis. We designated assessment times as Time 1 (T1) for the baseline admission RAI-MDS 2.0 assessment and as Time 2 (T2) for the first follow-up assessment at 90 days. Exposure variables were DNR and DNH directives based on the T1 RAI-MDS 2.0 assessment: 1) DNH present vs. not present and 2) DNR present vs. not present. Outcome variables were i) change in state of health instability at T2 versus T1, ii) location of care at T2 if discharged from the LTCH (hospital, another setting or home) and iii) death by T2. Discharge destinations and death are considered absorbing states, because transitioning to one of these states defines the end of the particular care episode in the LTCH. Residents remained in the model until the end of T2 or their first transition out of the LTCH.

We adjusted outcomes for age, sex, marital status, Activities of Daily Living Hierarchy Scale score, Cognitive Performance Scale score, diagnosis (binary variables for chronic obstructive pulmonary disease, pneumonia, diabetes, arthritis, renal failure, urinary tract infection, Alzheimer’s dementia and related dementias, heart failure, cancer and depression), day of stay, functional improvement potential, facility size, and province. We chose covariates on the basis of their expected associations with one or more of the outcomes states we modelled [3, 28, 29]. We only retained covariates that had significant associations with at least one outcome of interest in the models we used. We used odds ratio as a measure of association. To examine the degree of variation of this association according to illness severity, we looked at the computed odds ratios within the three levels of health instability as defined above: low, medium and high health instability. Analyses were performed using SAS Windows Server, version 9.4 (SAS Institute, Inc., Cary, NC).

## Results

### Baseline characteristics of LTCH residents

The final sample consisted of 123,003 LTCH residents (Table 2) who underwent 162,045 transitions. Overall, about 64% were women, and about 90% of patients were older than 75 years. Across Canada, 71% of residents had DNR directives, with the greatest proportion in Alberta at 83% and the lowest in Ontario at 70%. Over a quarter of residents had DNH directives with the highest proportion in Alberta (31%) and the lowest proportion in British Columbia (24%). Among residents with a DNR directive, 34.7% also had a DNH directive. Only 3.3% of residents with a DNH directive did not have a DNR directive.

**Table 2:** Distribution of baseline co-variates at time of admission to LTCH by DNH and DNR Status (*n* = 123,003 residents)

Covariate	Domain	Do Not Hospitalize			Do Not Resuscitate			All Residents (%)
		Not Present (%)	Present (%)	Χ^**2**^ ***p*** value	Not Present (%)	Present (%)	Χ^**2**^ ***p*** value	
**Age Group**	65–74	10,402 (78.3)	2889 (21.74)	< .0001	5602 (42.2)	7689 (57.8)	< .0001	13,291 (10.8)
	75–84	33,504 (76.9)	10,082 (23.1)		14,493 (33.3)	29,093 (66.7)		43,586 (35.4)
	85–94	42,105 (73.1)	15,503 (26.9)		14,270 (24.8)	43,338 (75.2)		57,608 (46.8)
	95+	5664 (66.5)	2854 (33.5)		1451 (17.0)	7067 (83.0)		8518 (7.0)
**Gender**	Female	58,889 (74.2)	20,529 (25.8)	< .0001	22,410 (28.2)	57,008 (71.8)	< .0001	79,418 (64.6)
	Male	32,786 (75.2)	10,799 (24.8)		13,406 (30.8)	30,179 (69.2)		43,585 (35.4)
**Marital Status**	Not married	65,784 (74.1)	22,942 (25.9)	< .0001	24,884 (28.1)	63,842 (71.9)	< .0001	88,726 (72.1)
	Married	25,891 (75.5)	8386 (24.5)		10,932 (31.9)	23,345 (68.1)		34,277 (27.9)
**Province**	AB	8541 (68.4)	3943 (31.6)	< .0001	2155 (17.3)	10,329 (82.7)	< .0001	12,484 (10.2)
	BC	13,276 (76.0)	4186 (34.0)		4856 (27.8)	12,606 (72.2)		17,462 (14.2)
	ON	69,858 (75.1)	23,199 (24.9)		28,805 (31.0)	64,252 (69.0)		93,057 (75.6)
**Diagnoses**	COPD	15,008 (73.8)	5339 (26.2)	0.0058	5403 (26.6)	14,944 (73.5)	< .0001	20,347 (16.5)
	No COPD	76,667 (74.7)	25,989 (25.3)		30,413 (29.6)	72,243 (70.4)		102,656 (83.5)
	Pneumonia	1561 (71.3)	628 (28.7)	0.0005	526 (24.0)	1663 (76.0)	< .0001	2189 (1.8)
	No pneumonia	90,114 (74.6)	30,700 (25.4)		35,290 (29.2)	85,524 (70.8)		120,814 (98.2)
	Diabetes	23,734 (76.7)	7212 (23.3)	< .0001	10,021 (32.4)	20,925 (67.6)	< .0001	30,946 (25.2)
	No diabetes	67,941 (73.8)	24,116 (26.2)		25,795 (28.0)	66,262 (72.0)		92,057 (74.8)
	Arthritis	25,744 (74.3)	12,372 (25.7)	0.116	13,145 (27.3)	34,971 (72.7)	< .0001	48,116 (39.1)
	No arthritis	55,931 (74.7)	18,956 (25.3)		22,671 (30.3)	52,216 (69.7)		74,887 (60.9)
	CRF	9965 (74.7)	3383 (25.3)	0.7262	3730 (27.9)	9618 (72.1)	0.0016	13,348 (10.9)
	No CRF	817,710 (74.5)	27,945 (25.5)		32,086 (29.3)	77,569 (70.7)		109,655 (89.1)
	ADRD	56,261 (73.8)	19,941 (26.2)	< .0001	21,548 (28.3)	54,654 (71.7)	< .0001	76,202 (62.0)
	No ADRD	35,414 (75.7)	11,387 (24.3)		14,268 (30.5)	32,533 (69.5)		46,801 (38.0)
	HF	13,754 (73.2)	5041 (26.8)	< .0001	4642 (24.7)	14,153 (75.3)	< .0001	18,795 (15.3)
	No HF	77,921 (74.8)	26,287 (25.2)		31,174 (29.9)	73,034 (70.1)		104,208 (84.7)
	Cancer	9587 (72.3)	3677 (27.7)	< .0001	3531 (26.6)	9733 (73.4)		13,264 (10.8)
	No cancer	82,088 (74.8)	27,651 (25.2)		32,285 (29.4)	77,454 (70.6)	< .0001	109,739 (89.2)
**CHESS**	0	46,724 (76.9)	14,062 (23.1)	< .0001	19,681 (32.4)	41,105 (67.6)	< .0001	60,786 (49.4)
	1	28,920 (73.7)	10,312 (26.3)		11,075 (28.2)	28,157 (71.8)		39,232 (31.9)
	2	11,482 (71.8)	4510 (28.2)		3790 (23.7)	12,202 (76.3)		15,992 (13.0)
	3	3428 (66.8)	1701 (33.2)		999 (19.5)	4130 (80.5)		5129 (4.2)
	4	1013 (62.8)	599 (37.2)		247 (15.3)	1365 (84.7)		1612 (1.3)
	5	108 (42.9)	144 (57.1)		24 (9.5)	228 (90.5)		252 (0.2)
Physician visit in last 14 days	Yes	72,875 (74.2)	25,302 (25.8)	< .0001	28,777 (29.3)	69,400 (70.7)	0.003	98,177 (79.8)
	No	18,800 (75.7)	6026 (24.3)		7039 (28.4)	17,787 (71.6)		24,826 (20.2)
CPS	0	11,197 (79.3)	2932 (20.7)	< .0001	4913 (34.8)	9216 (65.2)	< .0001	14,129 (11.5)
	1,2	33,974 (75.8)	10,873 (24.2)		13,413 (29.9)	31,434 (70.1)		44,847 (36.5)
	3,4	37,545 (73.4)	13,593 (26.6)		14,249 (27.9)	36,889 (72.1)		51,138 (41.6)
	5,6	8959 (69.5)	3930 (30.5)		3241 (25.2)	9648 (74.8)		12,889 (10.5)
ADLH	Scale = 0	5036 (80.4)	1228 (19.6)	< .0001	2145 (34.2)	4119 (65.8)	< .0001	6264 (5.1)
	Scale = 1,2	25,762 (77.0)	7698 (23.0)		10,590 (31.7)	22,870 (68.3)		33,460 (27.2)
	Scale > = 3	60,877 (73.1)	22,402 (26.9)		23,081 (27.7)	60,198 (72.3)		83,279 (67.7)

*AB* Alberta, *BC* British Columbia, *ON* Ontario, *COPD* Chronic Obstructive Lung Disease, *ADRD* Alzheimer’s Disease and Related Dementias, *CRF* Chronic Renal Failure, *HF* Heart failure, *CHESS* Changes in Health, End-Stage Disease, and Signs and Symptoms Scale, *CPS* Cognitive Performance Scale, *ADLH* Activities of Daily Living Hierarchy Scale

Table 3 shows absolute event rates by presence of absence of DNR and DNH directives. Both the presence of DNR and DNH directives were associated with fewer hospitalization and higher mortality at 90 days.

**Table 3:** Percentage of residents with DNR and DNH and who were acutely hospitalized or died within 90 days of admission to a LTCH (*n* = 123,003 residents)

	Do Not Resuscitate				Do Not Hospitalize			
	Not Present	Present	Difference	Χ^**2**^ *p* value	Not Present	Present	Difference	Χ^**2**^ *p* value
Hospitalized	23.2	18.8	−4.4	< .0001	22.0	14.5	−7.5	< .0001
Death	19.4	29.5	+ 10.1	< .0001	24.3	33.0	+ 8.7	< .0001

### Association between DNR and DNH directives and health transitions for LTCH residents at 90-day follow-up (T_2_)

Table 4 summarizes the association between DNR and DNH directives and subsequent health outcomes.

**Table 4:** Association between DNR and DNH directives and end-of-life outcomes for LTCH residents at 90-day follow-up (T_2_) by state of health instability at baseline

		End-of-life outcomes (T_**2**_) (adjusted odds ratio; 95% CI)^a^						
		Remained in LTC CHESS^b^ Score			Admitted to Hospital	Died	Discharged Other Setting^c^	Discharged Home
		0	1–2	3+				
Do Not Hospitalize (ref = Not Present)								
Health instability at baseline (T_1_)	0	–	1.04 (1.02–1.07)	1.10 (1.03–1.19)	0.67 (0.65–0.69)	1.48 (1.38–1.58)	0.92 (0.79–1.06)	0.91 (0.79–1.05)
	1–2	0.92 (0.90–0.95)	–	1.07 (1.03–1.12)	0.63 (0.61–0.65)	1.46 (1.40–1.52)	0.91 (0.81–1.04)	1.00 (0.86–1.17)
	3+	0.76 (0.68–0.85)	0.81 (0.76–0.87)	–	0.47 (0.43–0.52)	1.48 (1.37–1.60)	0.82 (0.60–1.11)	0.72 (0.44–1.16)
Do Not Resuscitate (ref = Not Present)								
Health instability at baseline (T_1_)	0	–	1.08 (1.05–1.11)	1.32 (1.21–1.45)	0.90 (0.87–0.92)	1.36 (1.25–1.49)	0.82 (0.72–0.94)	0.58 (0.51–0.65)
	1–2	0.91 (0.88–0.94)	–	1.19 (1.12–1.26)	0.82 (0.80–0.85)	1.38 (1.30–1.47)	0.85 (0.74–0.98)	0.55 (0.48–0.63)
	3+	0.75 (0.64–0.86)	0.85 (0.77–0.95)	–	0.63 (0.57–0.71)	1.11 (0.98–1.26)	0.76 (0.51–1.11)	0.53 (0.32–0.87)

^a^Multi-state transition models adjusted for: physician visits, age, gender, marital status, ADL Hierarchy scale score, Cognitive Performance Scale score, diagnosis (binary variables for COPD, pneumonia, diabetes, arthritis, renal failure, UTI, ADRD, heart failure, cancer, depression), facility size, data of stay, functional improvement potential)

^b^Changes in Health, End-stage disease, Signs and Symptoms (CHESS) scale is a measure of instability in health; higher scores indicate greater instability

^c^Other settings for transitions from nursing homes included discharges to other nursing homes, assisted living or retirement homes

#### DNH directives

The presence of DNH directives was associated with increased odds of transition to a higher health instability level while remaining in the LTCH for residents with low or medium baseline health instability. Similarly, the odds of transitions to better health were lower for residents with medium or high baseline health instability. The presence of DNH directive was also associated with increased odds of dying irrespective of baseline resident health instability.

The presence of DNH directives was associated with decreased odds of admission to acute care hospital overall. Residents with high baseline health instability had lower odds of admission to acute care (OR 0.47; 95% CI 0.43–0.52) than residents with medium and low baseline health instability (OR 0.63; 95% CI 0.61–0.65 and OR 0.67; 95% CI 0.65–0.69 respectively).

#### DNR directives

The presence of DNR directives was associated with increased odds of transition to a higher health instability level while remaining in the LTCH for residents with baseline low or medium health instability. As with DNH, the odds of transition toward improved health stability was lower with the presence of a DNR for the applicable baseline levels. The presence of a DNR directive was also associated with increased odds of dying for residents with low or medium baseline health instability; however, it was non-significant at the high instability level.

The presence of DNR directives was associated with decreased odds of admission to acute care hospital for the three health instability levels at baseline. It was also associated with decreased likelihood of discharge to another setting for residents with low or medium baseline health instability. However, in presence of a DNR directive, the odds of admission to acute care hospital for residents with high baseline health instability were significantly lower (OR 0.63; 95% CI 0.57–0.71) than the odds of admission to hospital for residents with low or medium baseline health instability (OR 0.82; 95% CI 0.80–0.85 and OR 0.90; 95% CI 0.87–0.92 respectively).

## Discussion

In this population sample of LTCH residents in 3 Canadian provinces, more than two-thirds of residents had a DNR directive and about 1 in 4 had a DNH directive. DNH orders were also frequently overturned. Both care directives were associated with greater health instability and death, but lower odds of hospitalization. The effect of both directives was greater among residents with the highest baseline health instability, primarily for worsening health instability and hospitalizations.

This manuscript is the fifth in a series of articles examining health transitions among LTCH residents and home care clients [2, 28–30]. Our findings that directives in LTCH are associated with lower odds of hospitalization support and further extend the generalizability of findings from recent literature [7, 8, 31]. Our use of multistate transitions models is better able than survival analysis to account for competing risks, and thus better able to model trajectories of change for multiple outcomes [2, 28–30]. Our findings also contribute to a greater understanding of the association between these directives to health instability, an outcome more specific to resident health than a health system outcome such as hospitalization.

The literature has shown that LTCH residents are frequently transferred to acute care hospitals at the end of life, a care episode from which many derive little benefit, and some may be harmed [2–4]. The intent of advance care planning is to properly understand the wishes of residents nearing the end of life, ensuring that these are well-informed with respect to the risks and benefits of interventions such as cardiopulmonary resuscitation and acute care visits. DNR and DNH directives are intended to capture the results of ACP discussions and support subsequent care planning. Our results and those of an older study by the Canadian Institute of Health Information confirm that the use DNR directives is common and has remained consistent over time [32]. The Canadian Institute of Health Information report also showed that DNR directives are rarely reversed in only 0.05% of residents. Taken together, these findings suggest that DNR directives are easily explained, universally understood, and sufficiently specific to be meaningfully adopted and implemented as part of an advance care planning process by LTCH teams, families and residents [32–34].

In contrast, our findings suggest that DNH directives have limitations. Even though they were associated with decreased rates of acute hospitalization, they were implemented only 26% of the time. Moreover, DNH directives were not associated with an absolute avoidance of hospitalization but with significant odds of being reversed, even among the residents with elevated baseline health instability and thus possibly at the end of life. Our findings, along with those of others who found no associations between a DNH directive and the odds of hospitalization, raise questions not only about the effectiveness of the DNH directive, but also about its acceptability and usefulness [33].

In our study, higher levels of health instability were associated with lower odds of being hospitalized among residents with either a DNR or a DNH directive in place, though these odds remain substantial. Higher baseline health instability is associated with greater odds of transition to a worsening health state, at which the risk of death is high regardless of whether a resident is hospitalized or not [2]. Other studies, however, have identified health instability as an independent predictor of hospitalization [35]. Taken together, these findings not only raise questions about whether resident wishes are fully respected during a health crisis, but also whether the advance care planning discussions that led to the DNH orders truly reflected well-informed resident wishes or were even person-centered. First, capable residents have the right to revise prior decisions about care and accept transfer to hospital for an acute illness [9, 36]. This right is recognized in some jurisdictions that require that all treatment decisions, such as whether to hospitalize or not, can only be made when they are required, and not ahead of time “just in case” [9, 37]. Second, despite previously expressed and informed resident wishes, hospital transfer may be appropriate for care of concerns for which the LTCH is unable or ill-equipped to address adequately, such as fracture management or urinary retention [9, 36]. Third, optimal approaches to ACP should always engage residents to elicit their perspectives and wishes, even though they may not be fully capable to decide [9]. We have previously shown that advance care planning conversations in Canadian LTCH often fail to sufficiently engage residents such that their wishes and goals are not fully expressed, nor understood by substitute decision makers or the care team [9, 38]. Moreover, residents of LTCH are often not provided with adequate information about their specific health context that would help them formulate and express care wishes [9, 38]. Finally, decisions may be related to institutional policies and practices. For instance, emergencies are often managed by on-call physicians who may not be familiar with the resident, have restricted access to advance care planning documentation, or face a distressed and unprepared family, and therefore err on the side of a transfer to an acute care hospital [9, 38]. Thus, while a DNH directive may be a consequential indicator of LTCH resident outcomes, it may not reflect adequate advance care planning or truly informed resident wishes. However, the CHESS scale, as a measure of a health instability that is routinely available upon admission to a LTCH, may represent important health context information to share with a resident or substitute decision maker when formulating care wishes.

This study has several strengths. First, the longitudinal cohort study design made it is easier to establish the existence of an appropriate time sequence. Second, the large sample size allowed to have less variability around the estimates and to make more reliable inferences. Third, the use of multistate transition models better accounts for competing risks and better describes resident health trajectories than regression models. There are a few limitations to consider. First, the data set did not include variables related to ethnicity, religious affiliation, facility ownership and operation, LTCH policies or practice that might shed further light on the results [39]. Similarly, we were unable to assess the quality and content of the advance care planning conversations that led to the documentation of DNH/DNR directives. Finally, additional prognostic factors that may explain some of the observed outcomes may not have been measured and considered in our analysis. However, we were able to examine a rich set of resident characteristics thus controlling for several confounding factors.

## Conclusion

Even though both DNH and DNR directives were more likely to be present for LTCH residents at risk of transition to greater health instability and death, DNH directives were present for only 26% residents and were frequently overturned, even for the most unstable residents. Our findings raise concerns that the DNH directive many be less aligned with resident wishes than it is reflective of a desired health service outcome. In light of our findings, and given emerging ethical concerns in some jurisdictions, we suggest that policies recommending DNH directives as a suitable indicator of resident-centered advance care planning be re-evaluated. Our findings also suggest the need to improve advance care planning in Canadian LTCH in a manner that enables residents to fully express their values and wishes in the context of their individual medical realities, supports person-centered decision-making at the point of care, and ensures that LTCH have the resources to deliver this care. The CHESS scale, as a measure of health instability, may be of value to inform advance care planning discussions with residents and their substitute decision makers.

## Data Availability

The datasets used and/or analysed during the current study are available from the corresponding author on reasonable request.

## Abbreviations

AB: Alberta
ADLH: Activities of Daily Living Hierarchy Scale
ADRD: Alzheimer’s Disease and Related Dementias
BC: British Columbia
CCRS: Complex Continuing Care Reporting System
CHESS: Changes in Health, End-Stage Disease, Signs, and Symptoms Scale
COPD: Chronic Obstructive Lung Disease
CPS: Cognitive Performance Scale
CRF: Chronic Renal Failure
DAD: Discharge Abstract Database
DNR: Do-not-resuscitate
DNH: Do-not-hospitalize
HF: Heart failure
LTC: long-term care
LTCH: long-term care home
ON: Ontario
RAI-MDS: InterRAI Resident Assessment Instrument-Minimum Data Set
